# Comparison of visual results and higher-order aberrations after small incision lenticule extraction (SMILE): high myopia vs. mild to moderate myopia

**DOI:** 10.1186/s12886-017-0507-2

**Published:** 2017-07-06

**Authors:** Hong-Ying Jin, Ting Wan, Fang Wu, Ke Yao

**Affiliations:** Eye Center, Second Affiliated Hospital, College of Medicine, Zhejiang University, Hangzhou, 310009 China

**Keywords:** Refractive surgery, Small incision lenticule extraction, Myopia, Higher-order aberrations, Complication

## Abstract

**Background:**

To compare the refractive results and higher-order aberrations (HOAs) after small incision lenticule extraction (SMILE) in high myopia and mild to moderate myopia patients.

**Methods:**

This prospective study included 165 eyes (86 patients) undergoing SMILE. According to the preoperative spherical equivalent (SE), treated eyes were divided into two groups: the high myopia group (more than -6.0 D, group-H) and the mild to moderate group (less than -6.0 D, group-M). Follow-up intervals were at 1 day, 10 days, 1 month and 3 months postoperatively. We obtained the following parameters: uncorrected (UDVA) and corrected distance visual acuity (CDVA), SE, efficacy and safety index, and HOAs.

**Results:**

Preoperative SE was -7.16 ± 0.93 D in group-H and -4.34 ± 0.97 D in group-M. At 3 months postoperatively, the SE in group-H and group-M was -0.20 ± 0.37 D and 0.01 ± 0.19 D (*t* = - 4.11, *P*<0.05), respectively. It was found that 77% and 98% had an UDVA of 20/20, 98% and 99% had a CDVA of 20/20 in group-H and group-M, respectively, while 87% and 95% had a SE within ±0.5 D and ±1.0 D in group-H, and 98% and 100% in group-M. The efficacy indexes were 0.98 ± 0.18 in group-H and 1.05 ± 0.10 in group-M (*t* = - 3.084, *p* < 0.05). The safety indexes were 1.06 ± 0.09 and 1.06 ± 0.09 (*t* = 0.153, *p* > 0.05), respectively. There were significant increases in total HOAs, 3^rd^-order coma, and 4^th^-order spherical aberrations.

**Conclusions:**

SMILE is an effective and safe surgery for correcting myopia. But the target correction amount in high myopia patients should be adjusted to avoid undercorrection and acquired more satisfaction. SMILE induced increases of HOAs.

**Trial registration:**

ChiTrial registration number: ChiCTR-OON-16009164. Retrospectively registered: 06.September.2016

## Background

Small incision lenticule extraction (SMILE) has been reported since 2011, for treatment of myopia and astigmatism [1, 2]. SMILE is a novel and less invasive technique because only a small incision is required and without a flap. Therefore, avoiding the creation of a flap and preserving more corneal nerve fibers, SMILE is expected to remedy the shortcomings of laser-assisted in situ keratomileusis (LASIK) and femtosecond laser-assisted LASIK (FS-LASIK) [3]. Studies have reported that SMILE minimizes dry eye, and maintains higher corneal sensitivity [4]. In addition, the postoperative corneal biomechanical strength is theoretically greater in comparison to LASIK and FS-LASIK [5–7]. Therefore, SMILE is considered to be a good selection mode for refractive surgery. There have been studies on SMILE techniques, but most reported results on visual acuity and refractive outcomes [8–10]. It is known that high-order aberrations (HOAs) are always responsible for postoperative symptoms, including halos, glare, monocular diplopia, and decreased contrast sensitivity after successful refractive surgery. Previous study showed that increased HOAs associated with LASIK. Recently, some papers reported that SMILE also induced HOAs [11–14]. However, there are limited numbers of studied comparing the induced HOAs of SMILE regarding the degree of myopia. The purpose of this study was to evaluate refractive predictability, efficacy, safety and HOAs for mild to moderate myopia and in high myopia.

## Methods

This is a prospective study, which included 165 eyes from 86 patients. All patients underwent SMILE surgery and completed 3 months follow up postoperatively were included. According to the preoperative spherical equivalent (SE), treated eyes were divided into two groups: the high myopia group (more than -6.0 D,group-H) and the mild to moderate group (less than -6.0 D, group-M). All patients underwent surgery at the Department of Ophthalmology, Second Affiliated Hospital, College of Medicine, Zhejiang University, from July to October, 2016. This research followed the tenets of the Declaration of Helsinki, and informed consent was obtained from the subjects after explanation of the nature and possible consequences of the study. Institutional review board approval was obtained for this study (No: 2016-025). The inclusion criteria included a minimum age of 18 years, no ocular or systemic diseases, stable refraction for at least one year, minimum corneal thickness of 480 μm, and minimum calculated residual stromal bed after a treatment of 280 μm. Patients who wore soft contact lenses were instructed to stop wearing them for at least one week. The two groups were compared with respect to the safety, efficacy, predictability, and HOAs of the SMILE treatment. The safety index (defined as postoperative CDVA/preoperative CDVA) and the efficacy index (defined as the postoperative UDVA/preoperative CDVA) were estimated [9].

### Surgical technique

The same experienced surgeon (H.Y.J.) performed all surgeries in the study. A VisuMax femtosecond laser system (Carl Zeiss Meditec AG, Jena, Germany) was used for surgical refractive corrections for all patients, with a repetition rate of 500 kHz and a pulse energy of 155 nJ. The spot and track distance were 4.5 μm for the cap and lenticule interface, 2.0 μm for the lenticule side cut and small incision. The exact details of the surgical procedure have been described previously by Sekundo et al [3]. The lenticule diameter was 6.5 mm, the cap diameter was 7.5 mm, and the intended cap thickness was 130 μm. The minimum lenticule side cut thickness was set at 10 μm. The optical zone diameter was equal to the lenticule diameter in patients with purely spherical refractive error. However, if the patient had astigmatism, a transition zone was added to convert the oval lenticule into a circle. The posterior surface of the refractive lenticule spiral in was created; the anterior surface of the refractive lenticule spiral out was formed. The side cuts made for access to the lenticule were set 120° apart at a width of 2 mm. The refractive lenticule was dissected by a spatula through the side cut opening incision and removed by a forceps. After surgery, all patients received a topical antibiotic for seven days and a topical steroid for two weeks. Hyaluronic acid lubricating drops were prescribed for more than four weeks. No adjustment to the manufacturer’s nomograms was done during the surgery.

### Postoperative evaluation

All patients were routinely examined postoperatively at 1 day, 10 days, 1 month, and 3 months. At each visit, uncorrected distance visual acuity (UDVA) and corrected distance visual acuity (CDVA) were measure in phoropter. Objective and manifest refractions, intraocular pressure, rotating Scheimpflug camera Pentacam system (Oculus GmbH, Wetzlar, Germany), and slit-lamp examinations were performed. Wavefront aberrations were measured with a Hartmann-Shack WASCA aberrometer (Carl Zeiss Meditec AG, Jena, Germany) with a 6.0 mm pupil using sixth orders Zernike polynomials. The root mean square (RMS) of total HOAs, spherical aberration, coma, higher-order astigmatism, trefoil, and tetrafoil were calculated. All postoperative complications were recorded.

### Statistical analysis

The main outcome measurements were the following: visual acuity, manifest refractions, RMS, and 3^rd^-order and 4^th^-order aberrations at 6-mm pupil size. Statistical analyses were performed using the SPSS software (ver. 18; SPSS, Chicago, IL, USA). All values are given as the mean ± standard deviation. The Kolmogorov–Smirnov test was used to test for normality. A paired-sample *t* test and ANOVA test was used for preoperative and postoperative comparisons. An independent-sample *t* test was used for comparisons between the group-H and group-M. Predictors for predictability were investigated using multiple linear regression analysis. Results were considered statistically significant at *p* < 0.05.

## Results

### Characteristics of eyes

All of the 86 patients attended the 1 day, 10 days, 1 month and 3 months follow-up examinations. Group-H and group-M included 62 and 103 eyes, respectively. The target refraction was emmetropia in all eyes in both groups. Preoperative characteristics of both groups are described in Table 1. No significant difference was evident between the two groups in age, intraocular pressure, or mean corneal power.

**Table 1:** Demographic and preoperative patient information (mean ± *SD and range)*

Parameter	Group-H	Group-M	*t*	*p*
Eye (n)	62	103		
Sex (M/F)	26/36	57/46		
Age (y)	23.32 ± 4.54 (18~32)	24.34 ± 6.12 (18~43)	-1.219	0.225
IOP (mmHg)	16.17 ± 2.54 (11~21)	16.06 ± 2.18 (11~22)	0.287	0.774
CCT (μm)	546.50 ± 22.99 (502~604)	549.80 ± 30.13 (489~618)	-0.792	0.430
Mean corneal power (D)	43.14 ± 1.39 (39.5~45.0)	42.94 ± 1.97 (39.5~46.0)	0.235	0.814
SE(D)	-7.16 ± 0.93(-6.00~-9.38)	-4.34 ± 0.97(-2.5~-5.88)	-18.304	0.000
Sphere (D)	-6.71 ± 0.91 (-5.75~-8.75)	-4.05 ± 0.96 (-2~-5.75)	-17.581	0.000
Cylinder (D)	-0.91 ± 0.60 (0~-2.5)	-0.60 ± 0.51 (0~-2.75)	-3.438	0.001
Lenticule thickness (μm)	123.71 ± 8.11 (107~138)	88.98 ± 14.35 (63~117)	19.852	0.000
Lenticule diameter (mm)	6.46 ± 0.19 (6.1~6.6)	6.56 ± 0.09 (6.1~6.6)	-3.847	0.000

*SD* standard deviation, *D* diopters, *CCT* central corneal thickness, *IOP* intraocular pressure Group-H high myopia group, Group-M mild to moderate myopia group

### Efficacy and safety

**Fig. 1:**
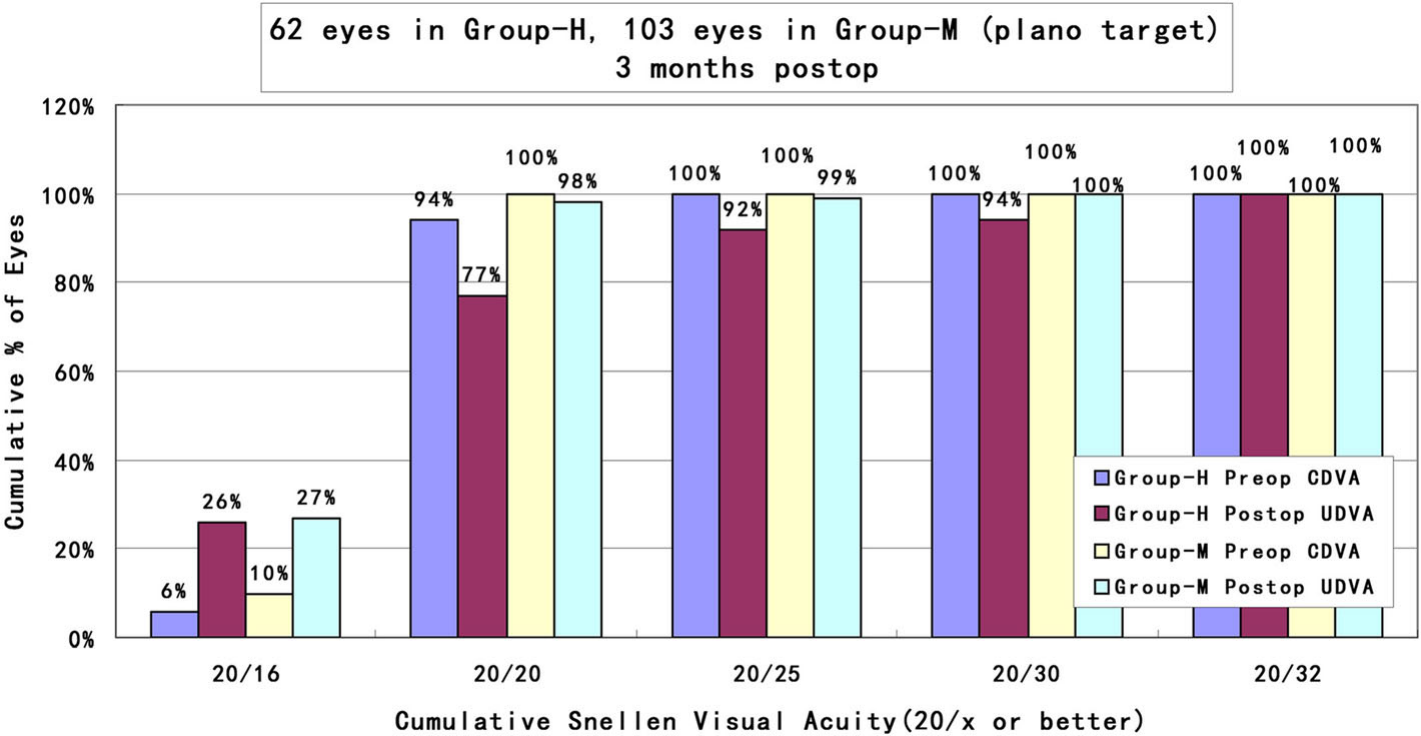
Cumulative percentage of preoperative CDVA and postoperative UDVA in Group-H and Group-M at 3 months follow-up (CDVA: corrected distance visual acuity; UDVA: uncorrected distance visual acuity; Group-H = high myopia group; Group-M = mild to moderate myopia group)

The comparisons of UDVA and CDVA after surgery between the two groups are shown Fig. 1, which illustrated the efficacy of the two groups by cumulative percentage of preoperative CDVA and postoperative UDVA at 3 months follow-up. The UDVA improved with the time after surgery in both groups. In total, 77% (48 eyes) and 98% (101 eyes) reached 20/20 or better at the 3-month follower-up in group-H and group-M, respectively. The efficacy indexes at 3 months were 0.98 ± 0.18 in group-H and 1.05 ± 0.10 in group-M, respectively. There existed a significant difference between the two groups (*t* = - 3.084, *p* < 0.05).

**Fig. 2:**
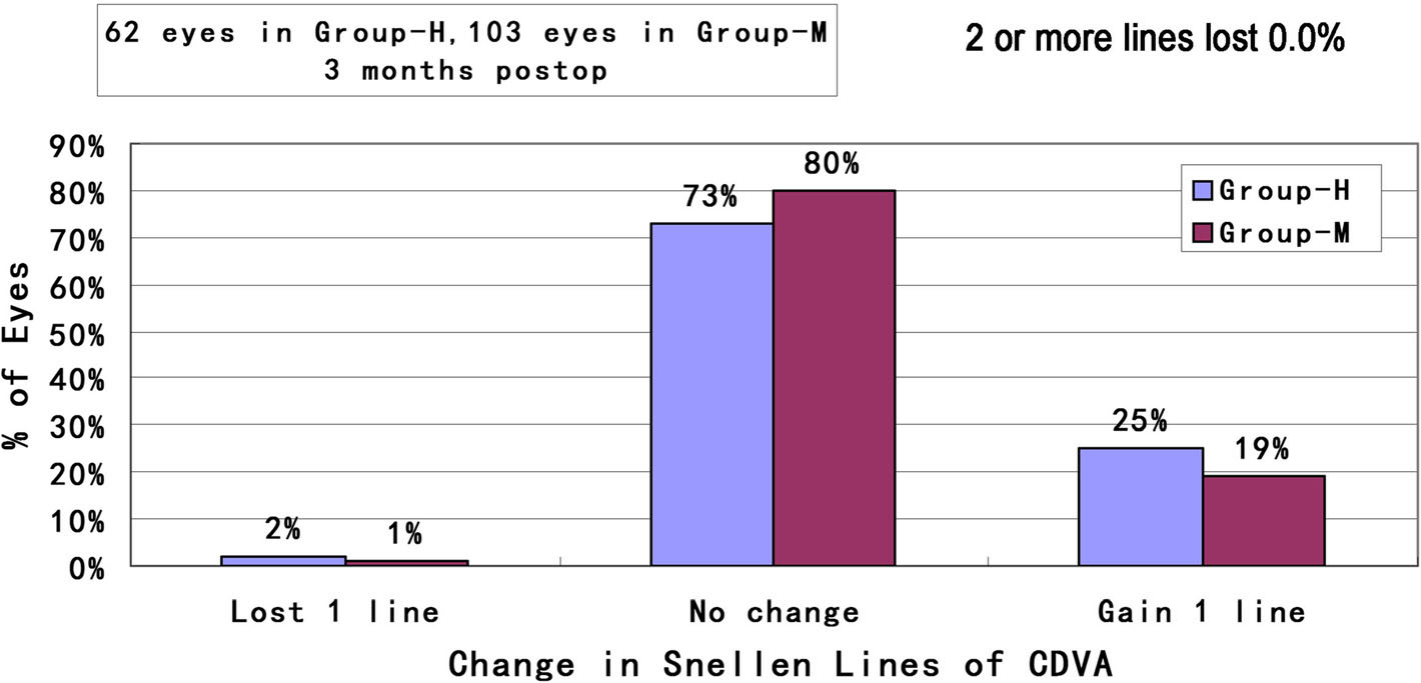
Change in CDVA in Group-H and Group-M at 3 months follow-up (CDVA: corrected distance visual acuity; Group-H = high myopia group; Group-M = mild to moderate myopia group)

Safety is illustrated in Fig. 2. The safety indexes at 3 months were 1.06 ± 0.09 in group-H and 1.06 ± 0.09 in group-M. There was no significant difference between the two groups (*t* = 0.153, *p* > 0.05). Postoperatively, two eyes in all patients lost one line of CDVA at the 3-month visit. However, 25% (16 eyes) and 19% (20 eyes) gained one line of CDVA in group-H and group-M, no change in 73% (45 eyes) and 80% (82 eye), respectively.

None of the patients had severe corneal complications. However, mild DLK (diffuse lamellar keratitis) was observed in four eyes at one day postoperatively. After using topical fluorometholone 0.1% ophthalmic solution, DLK dissolved quickly and did not have any effect on visual acuity. Suction loss occurred in three eyes. In these eyes, suction was reapplied successfully, and the fellow-eye procedure was performed as planned. All these eyes achieved a UDVA of 20/20 at the 3-month follow-up.

### Predictability and stability

**Fig. 3:**
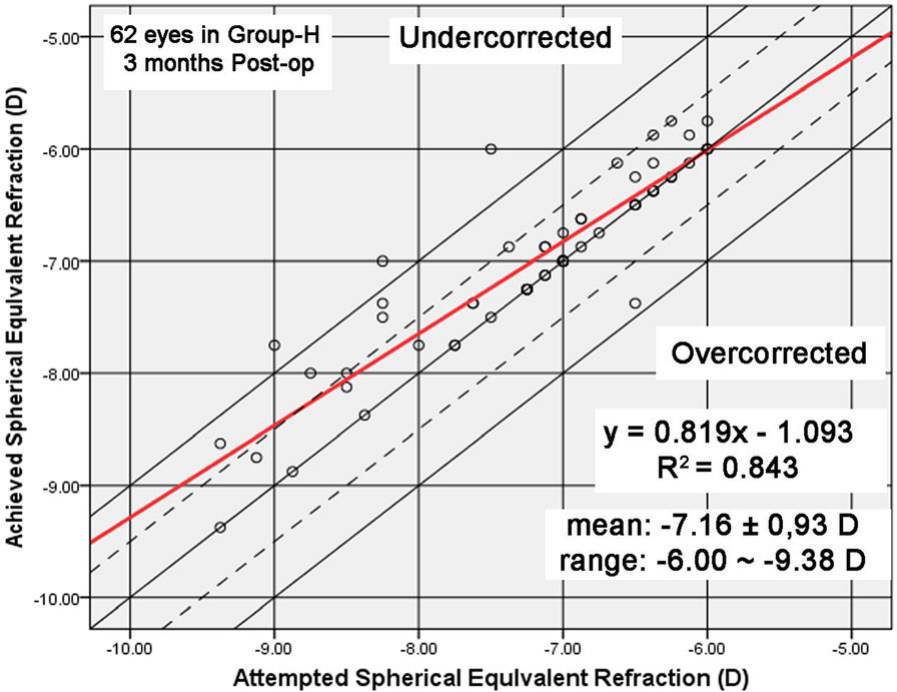
Achieved versus attempted change in SE at 3 months follow-up in Group-H (SE: spherical equivalent; Group-H = high myopia group)

**Fig. 4:**
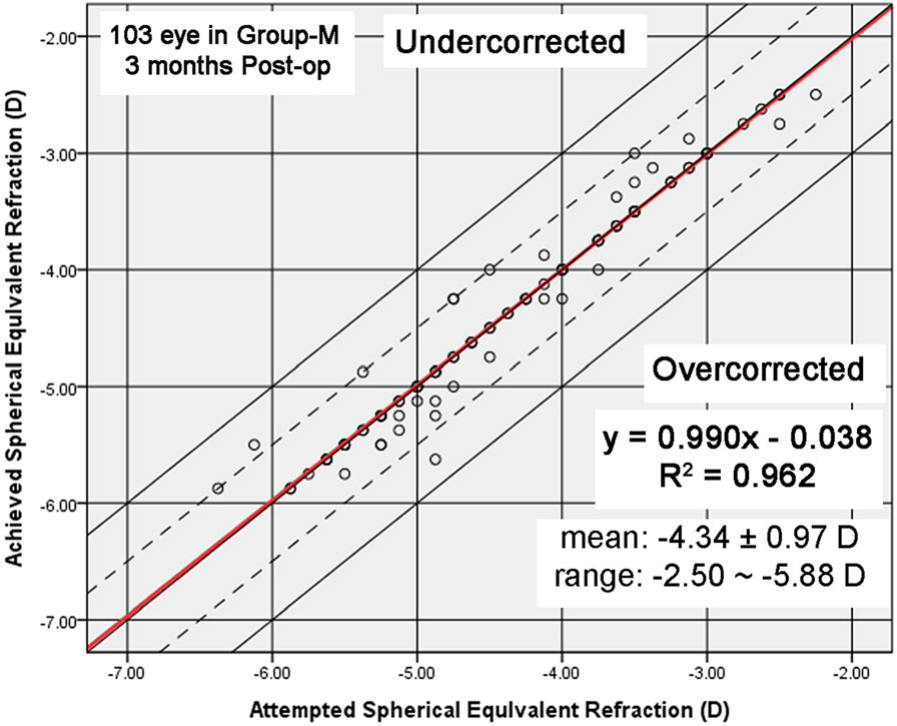
Achieved versus attempted change in SE at 3 months follow-up in Group-M (SE: spherical equivalent; Group-M = mild to moderate myopia group)

**Fig. 5:**
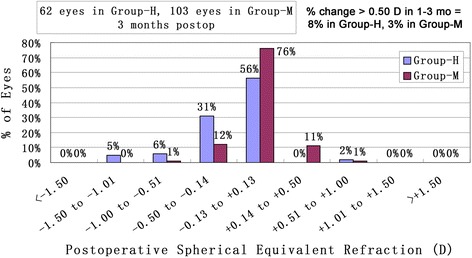
Accuracy of SE refraction in Group-H and Group-M at 3months follow-up (SE: spherical equivalent; Group-H = high myopia group; Group-M = mild to moderate myopia group)

**Fig. 6:**
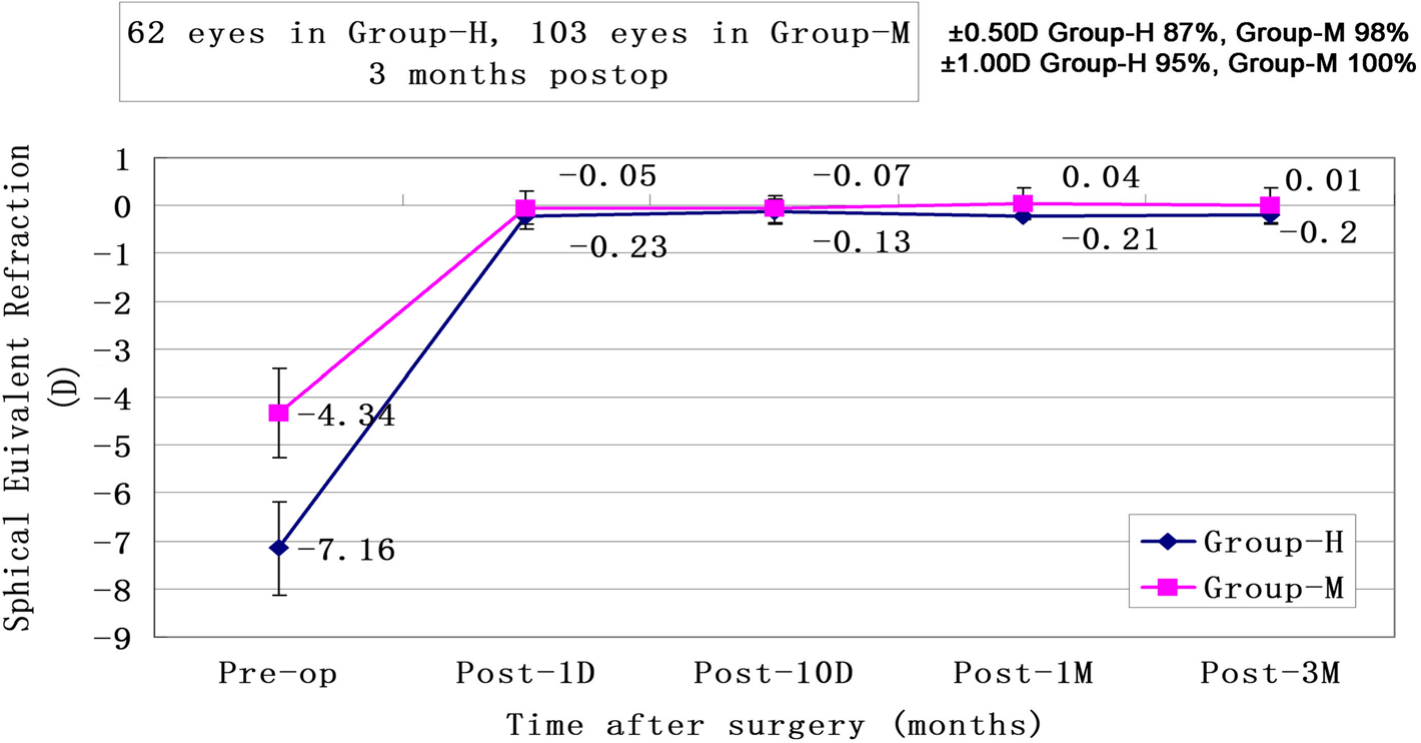
Stability of SE in Group-H and Group-M at 1 day, 10 days, 1 month and 3 months after surgery (SE:spherical equivalent; Group-H = high myopia group; Group-M = mild to moderate myopia group)

Figures 3 and 4 shows a scatter plot and linear regression analysis of attempted versus achieved spherical equivalent refraction at 3 months after surgery. Figure 5 illustrated the predictability of the surgery in both groups. At 3 months, 87% (54 eyes) and 95% (59 eyes) were within ± 0.5 D and ± 1.0 D of the intended correction in group-H, and 98% (101 eyes) and 100% (103 eyes) were within ± 0.5 D and ± 1.0 D in group-M, respectively. Figure 6 shows the mean postoperative SE of the two groups. At 3 months postoperatively, the SE in group-H and group-M was -0.20 ± 0.37 D and 0.01 ± 0.19 D (*t* = - 4.11, *P*< 0.05), respectively. Significant differences were also found between the two groups after surgery at 1 day, 10 days and 1 month after surgery (*p* < 0.05). There was no significant myopia regression at 3 months follow-up in either group.

### Higher-order aberrations

**Fig. 7:**
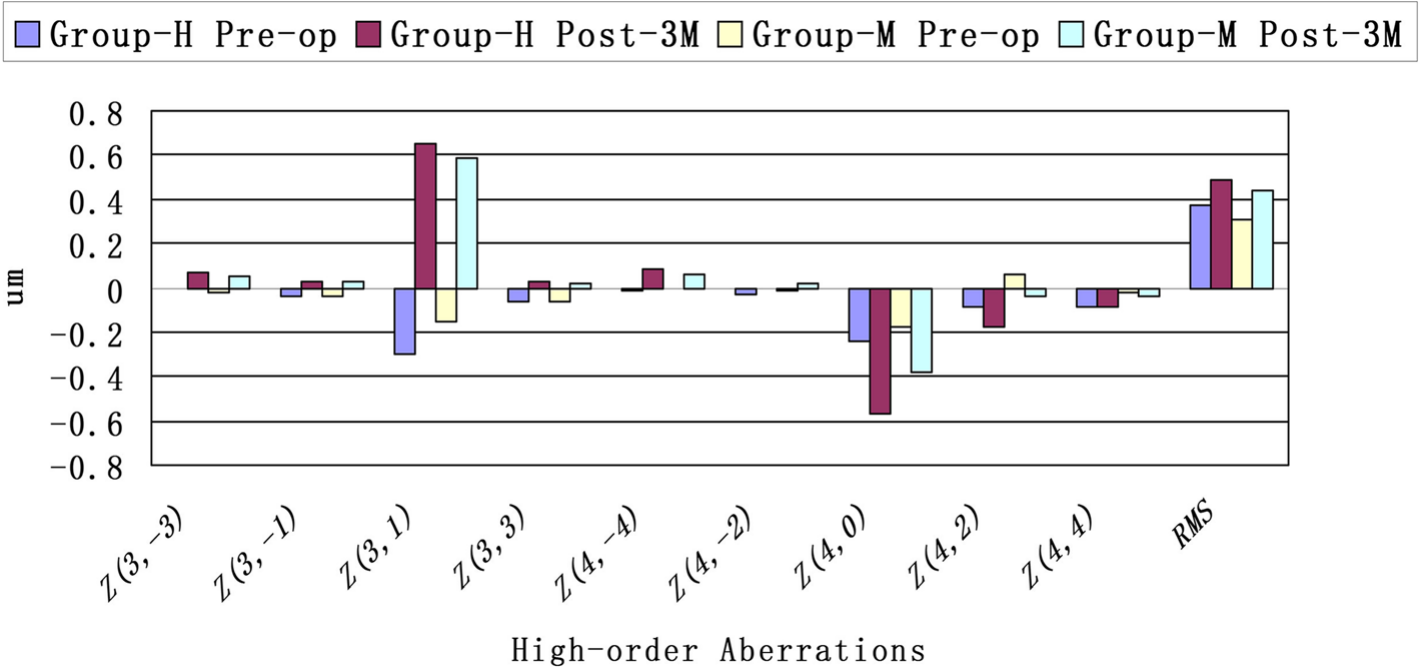
HOAs and RMS changes before surgery and at one and three months after surgery (HOAs : High order aberrations; RMS; Root mean square; Group-H = high myopia group; Group-M = mild to moderate myopia group)

In both groups, RMS value increased after SMILE, compared to the value before surgery. There were significant increases in postoperative 3^rd^-order horizontal coma, 4^th^-order spherical aberration, 4^th^-order oblique quadrafoil, and 4^th^-order vertical secondary astigmatism after surgery in both groups. The increase of spherical aberration was higher in group-H than in group-M. Table 2 shows the comparison of HOAs between group-H and group-M. RMS and HOAs changes are shown in Fig. 7.

**Table 2:** Comparison of aberration before and after SMILE surgery between group-H and group-M (mean ± *SD*)

Time	Pre-op			Post-3M		
Group	Group-H	Group-M	*p*	Group-H	Group-M	*p*
Vertical trefoil Z(3,-3)	0.00 ± 0.28	-0.02 ± 0.27	0.58	0.07 ± 0.34	0.05 ± 0.32	0.77
Vertical coma Z(3,-1)	-0.04 ± 0.21	-0.04 ± 0.27	0.94	0.02 ± 0.58	0.03 ± 0.45	0.88
Horizontal coma Z(3,1)	-0.30 ± 0.46^#♦^	-0.15 ± 0.43*^♦^	0.04	0.65 ± 0.41^#^	0.59 ± 0.56*	0.48
Oblique trefoil Z(3,3)	-0.06 ± 0.35	-0.06 ± 0.33	0.95	0.03 ± 0.31	0.02 ± 0.37	0.91
Oblique quadrafoil Z(4,-4)	-0.01 ± 0.12^#^	0.00 ± 0.13*	0.63	0.09 ± 0.12^#^	0.06 ± 0.12*	0.15
Oblique secondary astigmatism Z(4,-2)	-0.03 ± 0.12	-0.01 ± 0.14	0.37	-0.00 ± 0.13	0.02 ± 0.15	0.46
Spherical aberration Z(4,0)	-0.24 ± 0.26^#^	-0.18 ± 0.24*	0.13	-0.57 ± 0.32^#♦^	-0.38 ± 0.27*^♦^	0.00
Vertical secondary astigmatism Z(4,2)	-0.09 ± 0.20^#♦^	0.06 ± 0.19*^♦^	0.00	-0.18 ± 0.27^#♦^	-0.04 ± 0.24*^♦^	0.00
Vertical quadrafoil Z(4,4)	-0.09 ± 0.19	-0.02 ± 0.18	0.05	-0.08 ± 0.17	-0.04 ± 0.17	0.19
Total HOAs (RMS)	0.37 ± 0.14^#♦^	0.31 ± 0.12*^♦^	0.01	0.49 ± 0.16^#^	0.44 ± 0.15*	0.06

^#^Significant difference in HOAs at 3 months postoperatively compare with preoperatively (*p* < 0.05) in Group-H

*Significant difference in HOAs at 3 months postoperatively compare with preoperatively (*p* < 0.05) in Group-M

^♦^Significant difference in HOAs at equal time points between two groups (*p* < 0.05). HOAs high-order aberrations, RMS root mean square

## Discussion

In recent years, there has been a growing interest in SMILE as a new alternative refractive surgical option. Publications suggest that SMILE has excellent predictability, safety, and efficacy in correcting myopia and astigmatism [9–11, 15–18]. Here, we compare the three month SMILE outcomes between high myopia patients and mild to moderate myopia patients. It is a particularly accurate comparison because all of the SMILE surgery was performed by the same experienced surgeon. Moreover, we controlled surgical factors (e.g., laser energy setting) to precisely evaluate the efficacy, safety, predictability, and stability of SMILE. Also, we observed the HOAs changes after the SMILE surgery.

Regarding efficacy, UDVA improved gradually overtime after surgery in our study. Postoperatively, 77% of eyes in group-H and 98% in group-M had an UDVA of 20/20 or better at three months, respectively. The results were in accordance with results of other studies. Kim et al [16]. reported that 77% and 93% of eyes had 20/20 or better UDVA at 12 months in high and low to moderate myopia patients, respectively. Additionally, 80% to 96% of eyes were reported to have 20/20 or better UDVA at six months in low to high myopia patients in previous studies [15, 19, 20]. The higher success rate was seen in the mild to moderate group in our study.

For predictability and stability, the SE in group-H showed undercorrected. However, the SE in group-M was closer to the target refraction. At three months, 87% and 95% were within ± 0.5 D and ± 1.0 D of the intended correction in group-H, and 98% and 100% in group-M, respectively. There was no obvious regression in the three month follow-up time. In accordance with our results, Kim et al. [16] reported that 88% and 98% were within ± 0.5 D and ± 1.0 D in the high myopia patients and 88% and 97% in the low to moderate myopia patients at 12 months, respectively. Kim et al. suggested that SMILE surgery has a similar predictability, independent of the amount of myopic correction [16]. However, we suppose that the intended corrected myopia amount in high myopia patients should be revised in our future work, which will avoid the undercorrection in high myopia patient.

Regarding safety, two eyes lost one line of CDVA, 98% and 99% had a CDVA of 20/20 in group-H and group-M, respectively. However, different results were reported in other studies. Kim et al. [15] previously reported that 49% of eyes had an unchanged CDVA, 41% gained one line, 7% acquired two lines, 3% lost one line, and 0.3% lost two lines at six months. In another report of a one year follow-up by Kim et al. [16], 3% and 3% of eyes lost one line of CDVA, 37% and 43% were the same, 53% and 47% gained one line, and 7% and 6% of eyes gained two lines in mild to moderate myopia patients and in high myopia patients, respectively. In the report by Shah et al. [1], 4% of eyes lost one line, and 96% were unchanged or improved at six months. In the study by Sekundo et al. [20], 11% of eyes lost one line, and 89% were unchanged or improved at 12 months. This discrepancy may have resulted because our results were taken three months after surgery, which was a shorter follow-up time than in the other studies.

Some paper described the most frequent complication of the surgery, such as corneal haze, suction loss, small tear at the incision edge, cap perforation, difficult lenticule extraction [21], and residue of part of the intrastromal lenticule [22]. The incidence of suction loss in SMILE surgery was 4.4% in Wong et al. [23] and 2.1% in Osman et al [24]. In this study, suction loss occurred in three eyes of two patients (1.8%) during the small incision side cut procedure of the cap. After appropriate management, good visual outcomes were achieved. Other complications, such as epithelial ingrowths and haze, were not observed in this study.

HOAs contributed to the influence of visual quality after refractive surgery. Previous studies have shown that HOAs commonly increased after LASIK procedures. Recently, there have been some published studies on the induction of HOAs after SMILE [1, 12–14, 20, 25–27]. Shah et al. [1] found a significant increase in the RMS, higher-order coma aberrations, spherical aberrations, and 4^th^-order astigmatism, but there was no significant change in trefoil six months after SMILE. Sekundo et al. [20] observed that RMS, spherical aberration, and coma increased one year after SMILE surgery. Agca et al. [25] found that RMS, spherical aberration, coma, and trefoil increased after femtosecond lenticule extraction (FLEx) and SMILE surgery. Chen et al. [12] reported that a higher vertical coma was found in SMILE, and this was correlated to preoperative SE. Accurate centration during the SMILE procedure and controlling wound healing might be critical to minimize the induced coma. Yu et al [13] observed that the decentration displacement in SMILE was less than SBK surgery; however, vertical decentration would induce spherical aberration in SMILE surgery. Li et al. [27] demonstrated that the horizontal decentration induced horizontal coma, but the association between the magnitude of vertical decentration and the induced vertical coma tended to be nonexistent. In our study, the RMS and 3^rd^-order horizontal coma, 4^th^-order spherical aberration, 4^th^-order oblique quadrfoil, and 4^th^-order vertical secondary astigmatism increased significantly in both groups after surgery (*p* < 0.05). The magnitude of horizontal coma and spherical aberration are obvious (Fig. 7). The increase of spherical aberration was higher in group-H than in group-M. Han et al. [26] observed a significant increase of spherical aberration and coma after SMILE surgery, which did not decrease over the four years of follow-up. Among high order aberrations, postoperative coma was most affected and remained stable at all follow-up time points. In conclusion, the induction of spherical aberration is associated directly with the magnitude of the attempted diopters and ocular coma is associated with the magnitude of decentration. There exists varying conclusions may be due to the complicated influence factors, such as gravity, corneal irregularity, corneal haze, wound healing, amount of time following surgery, and intraocular pressure [27]. There maybe some relations with the corrected diopter, the position of cap rim cut, and the decentration ablation. However, the sample size of Group-H in the manuscript may not have sufficient statistical power (n=62) to detect differences. We used G-Power software (https://www.gpower.hhu.de/) to estimate the sample size. The statistical method is independent-samples T test. The α(the Type I error probability for a two sided test)was set to be 0.05, the power ( the probability of correctly rejecting the null hypothesis) was set to be 0.8, the effect size was set to be 0.5, and the allocation ratio (the ratio of control to experimental subjects) was set to be 1. And the results indicated that a total of 64 Group-H subjects and 64 Group-M subjects should be involved in our study. In our future work, the long-term changes of aberrations and a large sample size on SMILE still need further observation and discussion.

There were some limitations in this study. Firstly, this study included 165 eyes, and the available data covered only three months. A larger sample size and longer observation term were needed. Secondly, for bilaterally treated patients, both eyes were included, even though the two eyes of one patient are potentially correlated. This is a common mistake in ophthalmology research since the variance between eyes is usually less than that between subjects; the overall variance of a sample of measurements combined from both eyes is likely to be an underestimate of the true variance resulting in an increased risk of a Type 1 error [28]. Future research on the associations among visual quality, HOAs, and corneal biomechanics should be performed.

## Conclusions

In conclusion, our data indicates that SMILE is an effective and safe refractive surgical option. SMILE provides a predictable and stable correction of mild to moderate myopia. In high myopia patients, the intended correction should be modified, especially considering the age, occupation and dominate eye of patients. SMILE induced increases of HOAs. Further and larger studies on longer-term results are needed.

## Abbreviations

CDVA: Corrected distance visual acuity
FLEx: Femtosecond lenticule extraction
FS-LASIK: Femtosecond laser-assisted LASIK
HOAs: High-order aberrations
LASIK: Laser-assisted in situ keratomileusis
RMS: root mean square
SE: Spherical equivalent
SMILE: Small incision lenticule extraction
UDVA: Uncorrected distance visual acuity
